# Feasibility of sensory tongue stimulation combined with task-specific therapy in people with spinal cord injury: a case study

**DOI:** 10.1186/1743-0003-11-96

**Published:** 2014-06-06

**Authors:** Amanda E Chisholm, Raza Naseem Malik, Jean-Sébastien Blouin, Jaimie Borisoff, Susan Forwell, Tania Lam

**Affiliations:** 1 School of Kinesiology, University of British Columbia, Vancouver, Canada; 2 Department of Occupational Science & Occupational Therapy, University of British Columbia, Vancouver, Canada; 3 International Collaboration on Repair Discoveries, Vancouver Coastal Health Research Institute, Vancouver, British Columbia, Canada V5Z 1M9; 4 Centre for Applied Research and Innovation, British Columbia Institute of Technology, Vancouver, Canada

**Keywords:** Standing balance, Functional mobility, Rehabilitation, Robotic gait training, Sensory tongue stimulation, Spinal cord injury, Task-specific training

## Abstract

**Background:**

Previous evidence suggests the effects of task-specific therapy can be further enhanced when sensory stimulation is combined with motor practice. Sensory tongue stimulation is thought to facilitate activation of regions in the brain that are important for balance and gait. Improvements in balance and gait have significant implications for functional mobility for people with incomplete spinal cord injury (iSCI). The aim of this case study was to evaluate the feasibility of a lab- and home-based program combining sensory tongue stimulation with balance and gait training on functional outcomes in people with iSCI.

**Methods:**

Two male participants (S1 and S2) with chronic motor iSCI completed 12 weeks of balance and gait training (3 lab and 2 home based sessions per week) combined with sensory tongue stimulation using the Portable Neuromodulation Stimulator (PoNS). Laboratory based training involved 20 minutes of standing balance with eyes closed and 30 minutes of body-weight support treadmill walking. Home based sessions consisted of balancing with eyes open and walking with parallel bars or a walker for up to 20 minutes each. Subjects continued daily at-home training for an additional 12 weeks as follow-up.

**Results:**

Both subjects were able to complete a minimum of 83% of the training sessions. Standing balance with eyes closed increased from 0.2 to 4.0 minutes and 0.0 to 0.2 minutes for S1 and S2, respectively. Balance confidence also improved at follow-up after the home-based program. Over ground walking speed improved by 0.14 m/s for S1 and 0.07 m/s for S2, and skilled walking function improved by 60% and 21% for S1 and S2, respectively.

**Conclusions:**

Sensory tongue stimulation combined with task-specific training may be a feasible method for improving balance and gait in people with iSCI. Our findings warrant further controlled studies to determine the added benefits of sensory tongue stimulation to rehabilitation training.

## Introduction

There has been a great deal of interest on rehabilitation strategies such as task-specific training and sensory stimulation for facilitating neuroplasticity and enhancing motor recovery following neurological injury [1,2]. Task-specific training is built on the concept that motor output can be shaped and re-trained by relevant sensory cues, and involves a large number of repetitions based on motor learning principles [3]. For example, therapies that provide repeated practice of standing balance have been shown to be beneficial for people with incomplete spinal cord injury (iSCI [4,5]). As well, improved balance from task-specific training has resulted in better gait and functional independence for people with chronic stroke [6]. In people with SCI, improvements in mobility and ambulatory function are further associated with better health and social outcomes [7].

There is evidence that the effects of task-specific motor training can be further enhanced by sensory stimulation, as has been shown for gait disorders following stroke [8,9], hand function in people with stroke [10] or iSCI [11], and dysphagia following stroke [12]. Specifically in the SCI population, these effects include better upper extremity motor function and muscle strength [11]. One type of sensory stimulation is the administration of prolonged, tonic peripheral nerve stimulation at intensities high enough to recruit sensory nerve fibers but low enough to avoid activation of motor fibers. Although the stimulus activates only sensory fibers, corticospinal excitability can also be enhanced [13-15], consistent with concepts about the role of sensory input on motor output and learning throughout the central nervous system [16,17]. The somatosensory cortex may have an important underlying role in cortical reorganization after injury.

If sensory stimulation can enhance motor training outcomes, it might be possible that a higher volume of sensory input results in an additive effect [18]. For example, Conforto et al. showed that improvements in hand muscle strength were correlated with the intensity of somatosensory stimulation in individuals with stroke [19]. Enhanced stimulation volume could also be achieved by targeting different regions of the body. Perhaps the most sensitive area in humans is the tongue, which contains a high density of sensory receptors [20] with a large somatosensory cortical representation [21]. Somatosensory stimulation of the tongue can lead to changes in brainstem and cerebellum activation [22,23] in areas associated with the control of balance and gait [24,25]. Indeed, recent studies have demonstrated that sensory tongue stimulation can improve postural control in patients with balance disorders [26] and when combined with motor training, can improve balance and walking in patients with multiple sclerosis [27]. Sensory stimulation to peripheral nerves in other areas of the body may be difficult to regulate due to impaired sensation after iSCI (e.g. poor sensory perception and altered supraspinal sensorimotor interactions), whereas the tongue is usually not affected. These considerations make the tongue an inviting target for sensory stimulation in combination with task-specific training for the SCI population.

Thus, the primary purpose of this study was to test the feasibility of combining sensory tongue stimulation with balance and gait training on functional outcomes in people with iSCI. We present a case report of two individuals with motor-iSCI to show the feasibility as well as the potential effectiveness of this combined training approach with a lab-to-home based program on balance, functional ambulation, and quality of life in people with iSCI.

## Materials and methods

### Subjects

Two men with a motor-iSCI (American Spinal Injury Association [28]; ASIA C) due to trauma participated in this study after giving written consent. Subject 1 (S1) was 31-years old at 9.5-years post C5 level injury, while subject 2 (S2) was 30-years old at 12 years post T5-6 level injury. The body-weight for S1 and S2 was 65 kg and 72 kg, respectively. Subjects were able to ambulate over ground for at least 10 m unassisted with a wheeled walker and foot lifter (on the more affected side). However, both subjects relied on a power wheelchair for their daily mobility. Subjects had adequate range of motion to walk with the Lokomat robotic gait orthosis (Hocoma AG, Volketswil, Switzerland), and did not present with severe lower limb contractures or spasticity restricting passive range of motion. Neither of the subjects were participating in any formal rehabilitation program at the time of this study, and were free of other musculoskeletal or neurological conditions affecting mobility. All procedures were approved by the University of British Columbia and Vancouver Coastal Health Research Institute ethics committees.

### PoNS stimulator

**Figure 1:**
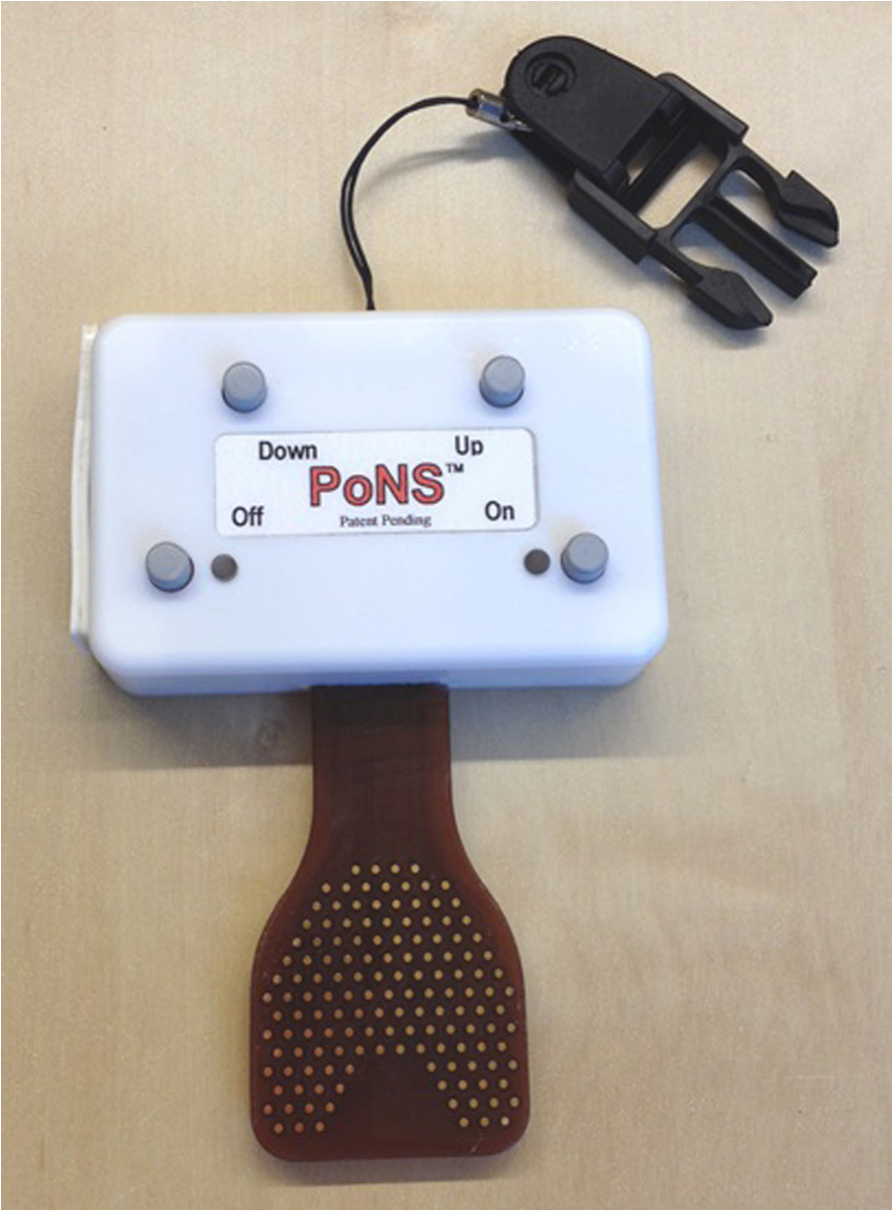
**A picture of the Portable Neuromodulation Stimulator (PoNS)™ used by the subjects during training.** The Up and Down buttons adjust the pulse width parameter to increase and decrease the stimulation intensity.

The Portable Neuromodulation Stimulator (PoNS)™ is a small electrode array (3 × 3 × 0.1 cm, and 100 g) that is held in place on the tongue’s surface with light pressure to the roof of the mouth (Figure 1). The stimulation consists of 19-V pulses delivered at a rate of 200 Hz with every fourth pulse removed [29]. The 143 electrodes are pulsed sequentially in groups of nine. Subjects were instructed to increase stimulation to a moderate-high level (pulse width adjustable from 0.4 to 60 μs) that was tolerable and not painful.

### Training program

**Figure 2:**
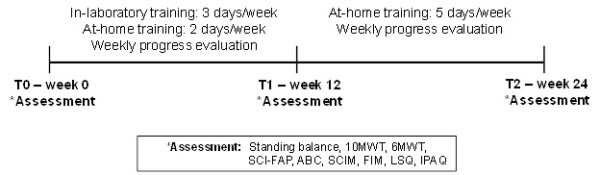
**A timeline of the training protocol and functional assessments.** The weekly progress evaluation included the 10 meter walk test (10MWT) and standing balance with eyes closed (and eyes opened for S2).

Subjects completed 12 weeks of balance and gait training (3 laboratory and 2 home based sessions per week) combined with sensory tongue stimulation using the PoNS [30], followed by an additional 12 weeks of home based training with the stimulator at 5 times per week (Figure 2).

In the lab, balance training consisted of four bouts of 5-minute of standing practice with 1-minute rest breaks between bouts. Subjects were instructed to focus on trying to stand for as long as possible with their eyes closed. Subjects wore a body weight support (BWS) harness for safety and to assist with upright standing. An easy BWS level was used as warm-up in the first bout; this was defined as the minimum BWS to maintain an upright posture for walking (e.g. no excessive knee flexion during stance or toe dragging). BWS was lowered by 10 kg for the second bout (difficult BWS level), and then raised by 5 kg (moderate BWS level) for the third and fourth bouts. Subjects were instructed to close their eyes when balanced with their hands off the parallel bars. Instructors provided verbal feedback on their performance and recorded the duration for which they could maintain their eyes closed with a stopwatch. Verbal feedback focused on different alignment issues in the lower and upper body on alternating days (e.g. shift weight evenly over both legs, keep a slight bend at the knee, keep heels on the ground, keep shoulders aligned with hips, etc.). Balance training was progressed between sessions by lowering BWS levels by 5 kg for each bout when 4 minutes of eyes closed could be consistently achieved in a single bout, and progressing to more challenging foot positions (e.g. narrow and tandem stance) when 0 kg BWS was achieved (Additional file 1).

Gait training consisted of six bouts of 5-minutes of walking with the Lokomat robotic gait orthosis, with rest breaks provided as required. The goal was to increase speed and decrease the amount of Lokomat guidance force on alternating sessions (e.g. if speed was increased, then force was held constant) for 30 minutes of continuous walking. BWS was set at the minimum level required to maintain an upright posture for walking (10 kg - S1 and 15 kg - S2). Subject’s reported their rate of perceived exertion (RPE) on the Borg CR10 Scale at the end of each bout [31]. If the RPE was < 5, walking speed was increased or Lokomat guidance force was decreased for the next bout by 0.1-0.2 km/h or 5-10%, respectively [32]. Gait training was progressed by using the previous session’s speed or force level reported with an RPE of 4 in the first bout for the next session.

For home-based sessions, subjects were instructed to practice balancing and walking for up to 20 minutes each using a walker at home or parallel bars at their local fitness gym for safety. Specific instructions for balance practice included keep eyes open, use tongue stimulator, and remember the verbal feedback tips provided during the laboratory sessions (see examples above). For walking practice, subjects were encouraged to limit rest breaks if possible and to walk at a moderate to fast pace. Subjects reported the duration of balance and gait training, and number of steps taken for gait training. An average and standard deviation was calculated for these parameters to describe the quantity of all home-based training sessions.

### Outcome measures

Static balance was assessed by recording the duration of time the subject could stand on a flat surface with eyes opened and closed with the feet positioned hip width apart. We also used the Activities-Specific Balance Confidence (ABC) scale to evaluate balance self-efficacy. Subjects rated their confidence in performing each activity (16 items) on a scale from 0 (no confidence) to 100% (complete confidence) without losing balance or becoming unsteady [33].

Walking function was evaluated by the 10-meter walk test (10MWT), 6-minute walk test (6MWT), and the Spinal Cord Injury-Functional Ambulation Profile (SCI-FAP). For the 10MWT, subjects walked along a 12-m walkway at the fastest speed they felt safe. Walking speed was calculated using the time required to traverse the middle 10 m, as measured by a stopwatch. For the 6MWT, subjects were asked to walk for 6 minutes at a self-selected speed around the edge of a gymnasium (25 m × 16 m), taking rest breaks if required. The total distance covered over 6 minutes was recorded. Both measures are valid and have excellent test-retest reliability (r = 0.983 and 0.981, respectively) in people with SCI [34,35].

Subjects also performed the SCI-FAP, a timed test of 7 walking tasks reflecting walking skills necessary for everyday mobility (e.g. obstacle crossing, stairs) [36]. The time required to complete each subtask is multiplied by a factor corresponding to the assistive device or level of manual assistance needed. The 7 sub-scores are then summed to provide a total score.

The Life Satisfaction Questionnaire (LSQ) was used to measure various aspects of overall life satisfaction that included 9 items on a 6-point scale [37]. Quality of life was assessed with the Impact on Participation and Autonomy Questionnaire (IPAQ), Functional Independence Measure (FIM) Activities and Participation, and the Spinal Cord Independence Measure (SCIM). The IPAQ focuses on the ability to participate in an activity and how their disability impacts their ability to participate, with 39 questions in 5 domains ranked from 0 (very good) to 4 (very poor) [38]. The FIM measures the level of a patient's disability and indicates how much assistance is required for the individual to carry out activities of daily living based on 15 items with scores ranging from 13 (lowest) to 91 (highest). The SCIM was developed specifically to evaluate self-care, respiration and sphincter management, and mobility for people with SCI, with a total score out of 100 [39].

Evaluations of static balance, walking function, and quality of life were conducted at 3 time points: pre-training (T0), after the initial 12-weeks of lab based training (T1), and follow-up (T2) after 12-weeks of home based training (Figure 2). We also assessed the duration of standing with eyes-closed (Additional file 1) and the 10 MWT every week to monitor progress throughout the training program. S2 was unable to stand with eyes-closed at the beginning of the study, so his duration of standing with eyes-open was also monitored weekly.

## Results

**Figure 3:**
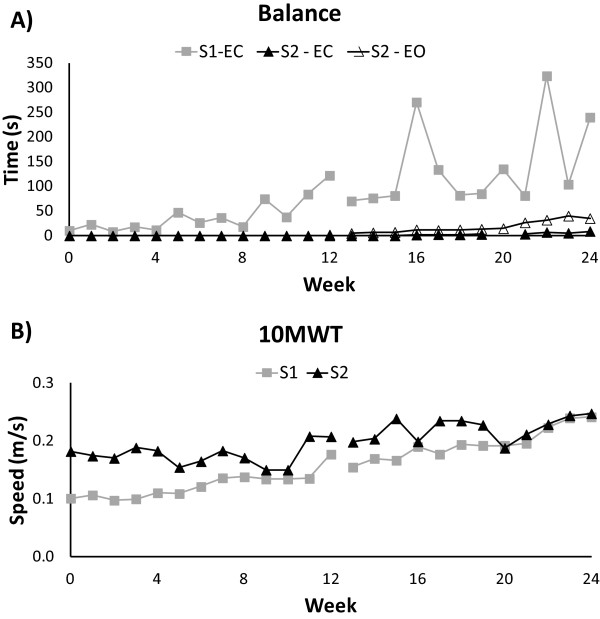
**Progression of A) balance and B) walking speed over the course of the laboratory-based (Weeks 0–12) and home-based (Weeks 12–24) training.** Balance was timed with eyes closed (EC) for both subjects, and eyes open (EO) only for S2. There was missing data at week 20 for S2 because balance with eyes closed was not attempted due to a headache and discomfort.

Weekly progression tracked during lab- and home-based training shows improved performance of balance with eyes closed and walking speed on the 10MWT (Figure 3).

### Laboratory-based training progression

Subjects completed 83% (S1) and 100% (S2) of the training sessions. S1 experienced a study-related skin abrasion due to friction from the Lokomat cuffs during gait training. Manual treadmill-training was conducted for the following 7 sessions to allow the injury to heal. For balance training, S1 started at 10 kg BWS (1st bout) and progressed to no BWS (all bouts) by the 20th in-lab session, and more difficult stance positions by the 24th in-lab session. During the 34th in-lab session, S2 progressed to trying no BWS from starting at 20 kg in the first session, and achieved 2.5 min of standing balance with eyes closed on the last session at 0 kg BWS. During Lokomat training, average treadmill speed increased by 0.5 km/h for both subjects, while guidance force contribution decreased by 37% (S1) and 24% (S2) by the end of the 12-week in-lab program.

### Home-based training progression

Subjects completed 86% (S1) and 88% (S2) of the home-based sessions, and reported that it was easy to train with the tongue stimulator at home. The average quantity of training within a session was 21 ± 5 minutes of balance and 16 ± 10 minutes of walking (234 ± 72 steps) for S1, and 17 ± 5 minutes of balance and 17 ± 6 minutes of walking (368 ± 121 steps) for S2.

### Balance outcomes

Weekly training outcomes for balance with eyes closed are displayed in Figure 3A. Standing balance with eye closed improved in S1 from 10.5 s at T0 to 122.1 s at T1 and 240.1 s at T2 (Table 1). S2 was unable to stand without support and eyes open at T0 and T1, but he could stand unsupported with eyes open for 35.5 s and eyes closed for 9.2 s by T2. ABC scores increased in both subjects from T1 to T2 appointments indicating greater confidence (Table 1).

**Table 1:** Summary of balance, gait and quality of life outcome measures

	**S1**			**S2**		
	**T0**	**T1**	**T2**	**T0**	**T1**	**T2**
Balance						
Eyes closed (s)	10.5	122.1	240.1	0	0	9.2
Eyes open (s)	24.0	>600	>600	0	2.2	35.5
ABC	— —	15.0	22.8	— —	21.3	31.0
Gait						
10 MWT (m/s)	0.10	0.18	0.24	0.18	0.21	0.25
6 MWT (m)	22.3	52.4	55.6	53.0	68.7	82.3
SCI-FAP	684.3	294.7	274.1	723.2	595.4	572.2
Quality of Life						
FIM	85	85	86	86	86	86
SCIM	66	77	78	81	81	82
LSQ	35.5	29	37	45	46	48
IPAQ	61	42	70	25	33	20

Note: T0, pre-training; T1, post-training; T2, follow-up after home-based training. The ABC scale was implemented at T1 because S2 was unable to stand supported with eyes open in the first 11 weeks of training. Lower SCI-FAP scores indicate better performance.

### Gait outcomes

**Figure 4:**
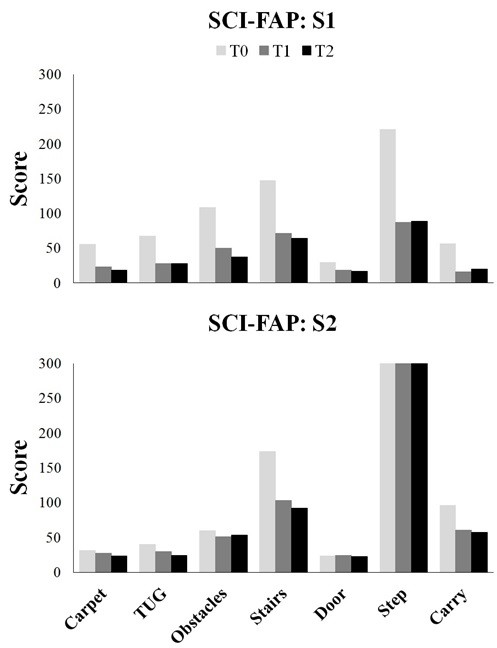
**The score for each task in the SCI-FAP test is plotted for each assessment (T0 - pre-training, T1 - post-training and T2 - follow-up).** The score represents the time to complete the task multiplied by a factor representing the amount of assistance required. Lower scores indicate improved functional ambulation. If the person cannot complete the task, the maximum score of 300 is assigned for that sub-task.

10MWT scores for weekly training outcomes are displayed in Figure 3B and performance on each task of the SCI-FAP is presented in Figure 4. 10MWT scores improved by 0.08 m/s and 0.03 m/s from T0 to T1, and 0.06 m/s and 0.04 m/s from T1 to T2 for S1 and S2, respectively (Table 1). Similarly, 6MWT scores improved by 30.1 m and 15.7 m from T0 to T1, and 3.2 m and 13.6 m from T1 to T2 for S1 and S2, respectively (Table 1). Total score on the SCI-FAP test improved by 410 and 151 points from T0 to T2 for S1 and S2, respectively, indicating better skilled walking function (Table 1). Figure 4 shows performance on the SCI-FAP for each task.

### Quality of life

FIM scores did not change from T0 to T1 (Table 1). SCIM scores increased from T0 to T2 by 12 points for S1, and by 1 point for S2 (Table 1). Slightly higher LSQ scores were reported at T2 compared to T0 for both subjects indicating greater satisfaction. IPAQ scores improved during the training from 61 at T0 to 42 at T1 and regressed up to 70 at T2 for S1, while S2 regressed from 33 at T0 to 25 at T1 and improved to 20 by T2 (Table 1).

## Discussion

This case study demonstrates three important findings; 1) the training program was feasible as subjects were able to safely complete at least 83% of the training sessions, 2) sensory tongue stimulation combined with task-specific training in persons with iSCI can improve balance and functional ambulation, and 3) these improvements were maintained for an additional 12 weeks with a home-based program. Also, subjects were able to maintain the tongue stimulator in position during training without any difficulty. Our results provide preliminary evidence in support of combining these rehabilitation strategies to improve balance and walking function in persons with chronic iSCI.

The results of this case study suggest that task-specific training with sensory tongue stimulation could improve balance as well as over ground walking speed and distance. Although we lacked a control group, the magnitude of some of the functional changes we measured here are comparable to the results of other studies in SCI. Standing balance with and without visual input improved over the training program, which corresponded with an increase of 7.8% for S1 and 9.8% for S2 on the ABC scale with 12 weeks of home-based training, indicating greater confidence to perform balance related activities. These changes are close to the minimal detectable change level in response to therapy at 11.1% on the ABC scale reported for the Parkinson’s disease population [40]. As we did not implement this measure until the end of the laboratory-based training program, it is possible that we could have captured greater changes in the ABC scale if it had been administered at T0. The improved performance on the 10 MWT in both subjects was also comparable to other studies reporting changes of 0.04 to 0.16 m/s after treadmill or over ground gait training [32,41-43], as was the change on the 6 MWT [43]. Further, S1 met the minimal detectable change level of 0.13 m/s for the 10 MWT [44]. Both subjects also exceeded the 92 points determined as 95% minimal detectable change on the SCI-FAP after laboratory-based training [45].

Our subjects continued to improve balance and walking function with an additional 12-week home-based program. In other studies with sensory stimulation, retention of functional gains over 1–2 months following the end of training has also been reported [8,46]. In a study by Field-Fote, participants who completed 12-weeks of manual-assisted body-weight support treadmill training (BWSTT) or functional electrical stimulation-assisted BWSTT maintained their functional improvements (e.g. 0.07 m/s faster gait velocity) even 6 months after the end of training [32]. However, in the same study, participants who trained with full assistance (100% guidance force) from the Lokomat did not retain improvements in gait speed [32]. In comparison, our subjects continued to show improvements (e.g. 0.04-0.06 m/s faster gait velocity) over 12 weeks with a home-based training program.

In the SCI literature, clinical studies have focused on rehabilitation strategies for improving gait, while less attention has been given to balance-specific training programs for standing [4,5]. Previous studies have demonstrated that people with iSCI can use sensory cues combined with motor practice to improve standing balance [4,5]. Sayenko et al. reported reduced fluctuations in the anterior-posterior and medial-lateral directions of the centre of pressure during 1 minute of standing with eyes closed, indicating improved postural stability after visual biofeedback training [5]. In comparison to these studies, our subjects’ initial standing balance ability was more impaired at the onset of training, as they were unable to stand unsupported for over 1 minute with eyes open. Although we did not measure centre of pressure, our findings reflect improved postural stability as the duration of balancing with eyes closed continued to increase throughout the training program. Results from the weekly progress evaluation of balance during the at-home phase of the program shows more variability in performance for S1, while a steady increase for S2. Examination of S1’s training logs did not yield any insights into possible reasons for this increased variability. The results also highlight important concepts that influence balance and gait performance, such as confidence and fear of falling. Both subjects initially scored low on the ABC scale indicating poor confidence in performing balance related activities without falling, but the scores improved after training. This emphasizes the potential clinical implications of our task-specific training program for individuals with severe balance impairments after iSCI to achieve meaningful gains in functional mobility. Since balance control plays an important role in performance of walking and daily activities [47], developing effective rehabilitation protocols to enhance balance practice may lead to improvement in other functional domains.

Functional ambulation refers to the ability to perform tasks that are frequently encountered in daily walking including walking on different surfaces, carrying objects, negotiating doors and obstacles, and ascending/descending curbs and stairs [36]. The lower scores on the SCI-FAP after training reflects improved ability to perform these tasks due to reduced time and amount of assistance required. Specifically, S1 demonstrated the greatest improvement in time on the obstacles, step, stairs and TUG tasks, while S2 was faster at the stairs, TUG and carry tasks. In addition, the ability to perform functional tasks with less assistance demonstrates improved dynamic balance, strength and locomotor control [35,48]. For example, both subjects were able to perform the sit-to-stand aspect of the TUG independently at post-training compared to the personal assistance they required at pre-training. These findings also suggest a change in strategy to perform the task more independently. Although S2 completed the obstacle task with no change in time at post-training, we observed that he was able to clear both obstacles with his less affected side with greater lower limb flexion rather than engaging compensatory movements and hitting the obstacle. Improvements on the SCI-FAP highlight important changes in strategy and independence of functional ambulation beyond the ability to walk faster after task-specific training combined with sensory tongue stimulation.

While the best rehabilitation strategy for gait training has been a debate (e.g. over ground vs. BWS treadmill training) [49,50], the optimum parameters for BWS treadmill training have not been established for individuals with SCI. Although the optimal dosage of the training program is beyond the scope of this study, in terms of frequency, duration and intensity, we demonstrated 5 days a week at 20–30 minutes of moderately intense training (RPE of 5) can improve balance and gait function. Our protocol used the RPE scale to determine the appropriate progression for speed and force contribution, reflecting the intensity of gait training. Our findings show the feasibility of using this approach to progress gait training on the Lokomat with the goal of increasing speed and reducing force contribution during 30 minutes of continuous walking. In addition, reducing BWS and changing stance positions effectively progressed the standing balance training. Furthermore, we demonstrated that 20 minutes of practice at home 5 days a week with sensory tongue stimulation could maintain or even facilitate continued improvements in balance and functional ambulation. The retention of our positive results and compliance rate with the home-based program indicates that this approach is a feasible option for people with chronic SCI.

Sensory stimulation techniques used during practice are thought to have an important role in cortical reorganization leading to the recovery of motor function after injury [18]. Afferent input may increase communication between the cortex and the corticospinal tract in people with SCI [13-15]. Stimulation of the somatosensory cortex may lead to increased efficiency in synaptic transmissions to the motor cortex, which appears to be important for motor learning [17,51]. Also, the sensory and motor cortices project to the cerebellum via the pontine nucleus, then send information back to the motor cortex [51]. Imaging studies have shown that somatosensory stimulation of the tongue leads to changes in brainstem and cerebellum activation [22,23]. In addition, post-tongue stimulation produced increased activity in the pontine region, likely from transmission via the trigeminal nucleus [22].

Although this case report provides important information regarding the potential benefit of combined task-specific training and sensory tongue stimulation after iSCI, there are several limitations to be considered. We only recruited two individuals because this was a pilot study to determine the feasibility of implementing the training program. Our outcome measures did not provide detailed insight on mechanisms of improved balance control or how changes in balance contributed to changes in other functional tasks. For future work, a more comprehensive balance assessment (e.g. biomechanical measures and/or dynamic tasks) may reveal specific aspects of balance control that improve with training. Due to the nature of a case report, it is impossible to discern how much of the observed adaptations were due to either the sensory tongue stimulation or task-specific training or to the combination of these two therapies. Future studies with larger samples are required to provide insight on whether combining the therapies results in an additive benefit.

## Conclusion

This case report describes our initial implementation of sensory tongue stimulation combined with task-specific training to enhance balance and gait functions in persons with iSCI. Our findings demonstrate the feasibility of incorporating sensory tongue stimulation with task-specific training to improve balance and gait for laboratory- and home-based programs for persons with iSCI. The clinical implications of this combined therapy protocol along with a continued home-based program to maintain improvements for balance and functional ambulation warrants further investigation.

## Abbreviations

6MWT: 6 minute walk test; 10MWT: 10 meter walk test; ABC: Activities-specific balance confidence; ASIA: American Spinal Injury Association; BWS: Body-weight support; FIM: Functional independence measure; IPAQ: Impact on participation and autonomy questionnaire; iSCI: incomplete spinal cord injury; LSQ: Life satisfaction questionnaire; PoNS: Portable neuromodulation stimulator; RPE: Rate of perceived exertion; SCI-FAP: Spinal cord injury-functional ambulation profile; SCIM: Spinal cord independence measure.

## Competing interests

The authors declare that they have no competing interests.

## Authors’ contributions

All authors contributed to the concept and project design. AC and TL provided writing of the manuscript. JSB, JB, SF and RM provided review of manuscript before submission. AC and RM conducted the training and data collection. TL provided the facility and equipment. TL, JSB, JB and SF completed the grant application to fund the project. All authors read and approved the final manuscript.

## Supplementary Material

Additional file 1 This video shows our balance assessment with eyes closed at pre-training (T0) and after the initial 12 weeks of training (T1), along with our in-laboratory balance training set-up. Click here for file
